# Gender, Physical Self-Perception and Overall Physical Fitness in Secondary School Students: A Multiple Mediation Model

**DOI:** 10.3390/ijerph17186871

**Published:** 2020-09-20

**Authors:** Pedro Jesús Ruiz-Montero, Oscar Chiva-Bartoll, Antonio Baena-Extremera, David Hortigüela-Alcalá

**Affiliations:** 1 Department of Physical Education and Sport, Faculty of Education and Sport Sciences, Campus of Melilla, University of Granada, 52071 Melilla, Spain; pedrorumo@ugr.es; 2 Department of Education and Specific Didactics, Faculty of Humanities and Social Sciences, Universitat Jaume I, 12071 Castellón, Spain; 3 Department of Education Sciences, Faculty of Education, Universitary Campus of Cartuja, University of Granada, s/n, 18071 Granada, Spain; abaenaextrem@ugr.es; 4 Department of Specific Didactics, Faculty of Education, University of Burgos, 09001 Burgos, Spain; dhortiguela@ubu.es

**Keywords:** self-confidence, physical capacity, body image, sport ability, strength perceived, self-esteem

## Abstract

*Background:* Physical self-perception is often related with better physical fitness perception in adolescents. Moreover, it is an important social cognitive perspective to provide suitable mental health in this population. However, this relationship is unequal between boys and girls. The physical fitness is a marker of health in young population. The aims of the present study were the following: (1) to compare physical self-perception and self-reported overall physical fitness (OPF) between boys and girls (gender) and body mass index (BMI) status, and (2) to determine the mediating role of all physical self-perception subscales (except physical condition) and BMI status in the link between gender and OPF in adolescent students. *Methods:* This cross-sectional study consisted of 85 adolescent students of secondary school between 12 and 17 years of age; 41 were boys (M_age_ = 14.6, SD = 1.7) and 44 were girls (M_age_ = 14.4, SD = 1.6). Adolescent participants completed all clinical characteristics by body composition measures (age, body weight, body height, and BMI). Physical self-perception was assessed by the physical self-perception profile (PSPP) whereas the international fitness scale (IFIS) was used to predict the self-reported OPF of adolescents in the present study. *Results:* Gender (boys and girls) differed significantly in all PSPP subscales and OPF, whereas the BMI status (underweight = 19 students, normal weight = 53 students, overweight/obese = 13 students) showed significant differences in all clinical characteristics, physical condition (PSPP), and OPF. A multiple mediation analysis was performed using bias corrected bootstrap. This multiple mediation analysis revealed that all PSPP subscales were significant mediators between gender and OPF: attractive body (*p* = 0.013), sport competence (*p* = 0.009), physical strength (*p* = 0.002), and self-confidence (*p* = 0.002). The total direct effect of gender on OPF was significant (*p* = 0.002). Moreover, the multiple mediation estimated a completely standardized indirect of X on Y for attractive body (effect = 0.109), sport competence (effect = 0.066), physical strength (effect = 0.130), and self-confidence (effect = 0.193). *Conclusions:* These findings contribute to understanding the link between gender and OPF in adolescent students and the mediation of physical self-perception and OPF in this relationship. In addition, strategies focused to improve self-confidence and physical self-perception are necessary in female adolescent students, because boys showed better physical self-perception in all PSPP subscales. Girls are a risk group because they report low physical self-confidence with their respective insecurity feelings and psychological disorders. Thus, personal physical self-perception must be considered as an important social cognitive perspective to provide suitable mental health in children and adolescents.

## 1. Introduction

Practice of physical activity (PA) and sport helps students during physical education (PE) lessons and non-scholar time to acquire better feelings of personal satisfaction [1], PA motivation [2], and better self-perception of physical fitness (PF) [3]. Self-perception is a reflection of the student about their capacity to meet the physical limits in PA and sports [1]. According to Fox et al. [4], physical self-perception is the main characteristic of the search for mental health and well-being. On the other hand, self-perception of PF is considered to be multidimensional, composed by perceived PF and athletic competence in sport activities and activities with component of muscular strength, flexibility or cardiorespiratory fitness [5]. Moreover, perception of the own PF is sensitive to variations in PA levels and psychological self-satisfaction (e.g., body-image, satisfaction during PA practice, insecurity, etc.) [5,6]. Self-reported PF in children and adolescents is useful to establish possible cardiovascular disease risk and diverse levels of PF in this population [3,7]. Thus, personal physical self-perception assessed by validated instrument could be considered as an important social cognitive perspective to provide suitable mental health in children and adolescents [4,7]. Moreover, self-perception of physical fitness in youth is related with a positive identity and less behavior disorders when they practice PA [8].

Adolescent students’ behaviors during a class or their leisure time may vary depending on a number of factors, such as sport motivation, acceptation of their self on sport activities or the satisfaction of physical competence by physical self-perception [9]. It considers that the correct physical self-perception strongly influences teenager and adolescent motivation and the low or high control of self-perception behaviors, and is the reason for the proliferation of studies in this regard [10,11]. The physical self-concept shows a relative importance because it is based on the relationship between the individual’s personal beliefs and their subsequent behavior.

Fox and Corbin [12] included five physical competences regarding to the physical self-perception in their instrument physical self-perception profile (PSPP) with the purpose to analyze the effects of the relationship between physical´s perception and participation in PA and sport activities: physical condition, attractive body, sport competence, physical strength and self-confidence.

In this sense, research conducted with young participants have found that a better perception of one physical condition is related with the practice of regular physical exercise and sport in this population [5]. In addition, PF is associated with higher motivation towards PA and better well-being, especially when existing a mix of educative activities such as physical and nutritional education or with the promotion of coeducation [10]. However, previous studies have shown that boys perceive better physical condition than girls because of the high satisfaction in everything related to body perception and PA inside and outside of the school context [1].

The next PSPP physical competence is the attractive body measure. This PSPP subscale is controversial because the body representation has been identified very often with psychiatric disorders and dissatisfaction in several type of population [13,14], specifically in girls [9,15]. The adolescence is characterized by a slow progression from the puberty to adulthood with biological, psychological, social and cognitive changes that varies according to gender and age where the self-image attitude is very significant [16]. Body image has been based overall by self-report body size judgement [17] and the girls are often more critical and demanding than boys about her body image due to social pressure and the respective obsession with perfection [18].

Sport activities for adolescents offer multiple possibilities to improve personal and interpersonal skills, and therefore a better sport competence [1]. On the other hand, girls´perceptions of social competence are not as high as the boys´ perception in sport practice [19] and this fact must be taken into account in gender comparative studies.

Another PSPP subscale is physical strength which is strongly associated with general self-concept, happiness and life satisfaction [20,21,22]. In this line, physical strength self-perception has shown to be associated with general fitness in younger population [23], specifically with boys for the obsession to show the muscularity of these [24]. Self-confidence and social identity respect to the sport participation´s interest for adolescent could vary with the time and it is important to begin the contact with sport activities from an early age to stablish a natural relationship and a strong self-confidence in the young practitioner [19]. Self-confidence in boys is normally higher than in girls because they like to be more active in sports and every activity of their daily life [25].

After all literature cited previously regarding to the evidence of the physical self-concept importance in youth, we highlight that physical self-perception [26] or body mass index (BMI) status [27] are markers of health in this population. Therefore, both self-perception through PSPP subscales and BMI status can play an important role as mediators between gender and overall physical fitness (OPF) in adolescents. Regarding to BMI status in students of secondary school between 11–14 years old, the daily life-style and several aspects of the PA practice such as interest for sport practice, frequency or aptitude are related to different BMI status as overweight [28]. Differences of BMI status between adolescent boys and girls is difficult to appreciate due to the continuous body composition changes during this period [25].

PF can be objectively or subjectively measured. There are multitude of PF objective measuring with physical tests focused on adolescents such as the ALPHA-Fitness test (Assessing Levels of Physical Activity) [29], the HELENA study (Healthy Lifestyle in Europe by Nutrition in Adolescence) [30], and the muscular strength measuring test [31], etc. On the other hand, it is possible to use subjective questionnaires to measure the PF such as the international fitness scale (IFIS) [3,7] or self-reported cardiorespiratory fitness [32]. However, objective measurements are always more expensive and difficult to perform with participants than subjective measurements as questionnaires or written tests. The last one is easily possible by the IFIS [7] with a self-report measure for youth to identify the level of physical fitness according to five components: OPF, cardiorespiratory fitness, muscular strength, speed/agility and flexibility.

**Figure 1:**
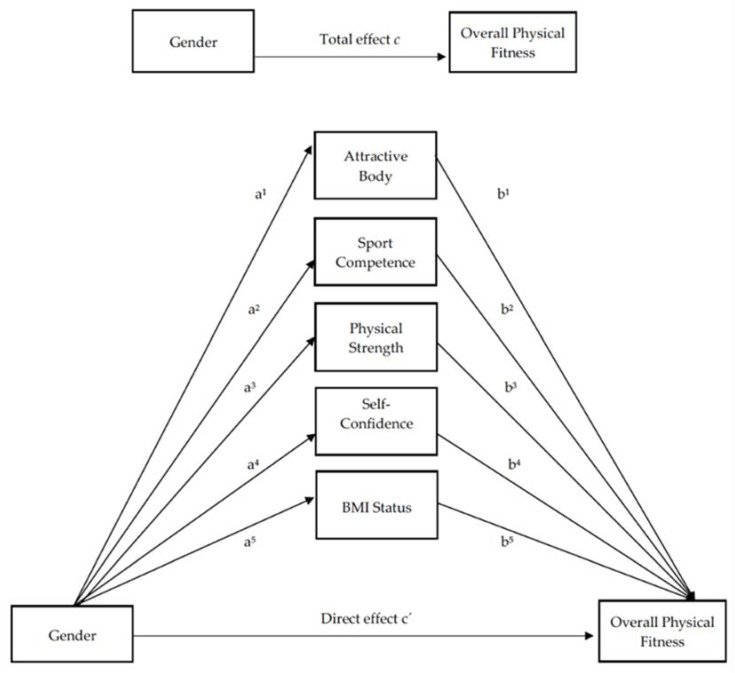
Hypothetical model of the relationship between gender and overall physical fitness.

Taking into account all of the above, the main aims of this study are (1) to analyze the difference of physical self-perception and self-reported OPF between boys and girls (gender) and BMI status (underweight, normal weight, overweight/obese), and (2) to determine the mediating role of all PSPP categories (except physical condition) and BMI status in the link between gender and OPF in adolescent students (Figure 1).

## 2. Materials and Methods

### 2.1. Participants

A cross-sectional design was used in this study with an observational and descriptive perspective. The sample selection process used was non-probabilistic and convenient. The total sample was composed of 85 adolescent students with ages ranging between 12 and 17 years old (M_age_ = 14.5, SD = 1.6). According to the profile of the participants in the present study, 41 were boys (M_age_ = 14.6, SD = 1.7) and 44 were girls (M_age_ = 14.4, SD = 1.6). Both student groups were recruited from a secondary school in a village in Granada province, Andalusia region. This educative context was representative of that town because it was the only secondary school of the village. A total of 140 students from four classrooms were invited to participate but only 85 accepted to be part of this study (60.7% of the total). There were not any dropout and all participants finished the study.

An inclusion criterion for adolescent students in this study, was to have no illness limitation as measured by a bioelectrical impedance analysis and no history of neuropsychological impairment that could affect the results of the experiment. All variables obtained were subjective (questionnaires) except for clinical characteristics. All participants were selected for the study through information advisor of center, read and signed an informed consent statement before taking part in the study. The participants were fully debriefed about the purpose of the study at the end of experiments. The wide status of age and number of participants is explained because of the non-obligatory nature of the study.

Considering a statistical power of 80% (z), a type 1 margin of error or alpha of 0.05, a response distribution of 50% (r) and sample population of young people in the period of secondary school (12–17 years) (N = 184) in the village, the sample size of the present study was in the recommended range. The following formulas were used [33]:

x = Z(c/100) 2r (100 − r)

n = N x/((N − 1) E2 + x)

E = Sqrt [(N − n) x/n(N − 1)]

### 2.2. Research Design

The present study shows the physical self-perception as a daily condition in the life of adolescents. Thus, we try to analyze the impact of gender (boys and girls) on the OPF self-perception and the results of this relationship. However, it is necessary to highlight more specific physical self-perceptions (mediators) that help us to understand better this relationship between gender and OPF (Figure 1). These physical self-perceptions are attractive body, sport competence, physical strength and self-confidence (all, PSPP subscales). In addition, the BMI status is not a physical self-perception but is a health marker that affects to the association between male or female adolescents and the OPF [28]. Therefore, we decided to include BMI status as the fifth mediator between the association of gender and OPF.

### 2.3. Measurements

Clinical characteristics such as body weight (kg) and body mass index (BMI)(kg/m^2^) were measured by bioelectrical impedance analysis with a Tanita SC 330s. With the intention of specifying groups within BMI reference criteria of World Health Organization (WHO) (https://gateway.euro.who.int/en/indicators/mn_survey_19-cut-off-for-bmi-according-to-who-standards/) [34], all BMI status (underweight, normal weight, overweight/obese) were calculated to relate with clinical characteristics, physical self-perception profile, and self-reported physical fitness. Body height (cm) was measured using a stadiometer (Seca 22, Hamburg, Germany). In addition, all participants completed a physical self-perception profile (PSPP) questionnaire and a self-reported physical fitness assessment by IFIS.

Physical self-perception profile (PSPP) [12] was used in the Spanish version [9] to measure the physical self-confidence of adolescent students in this study. The PSPP examines the students’ PA and sports practice in their daily life, as well as their sports habits in their leisure time, aiming to better understand the physical self-concept of these students. If the physical capacity perception is more positive, the levels of participation in PA are probably going to increase in children and young people [35]. A total of 30 items make up this questionnaire, and they are grouped in five subscales [9,12]: physical condition (physically active, physical performance, etc.), attractive body (confidence in body image, maintain an attractive body), sport competence (ability to learn sports, sportsmanship, sport confidence), physical strength (confidence of one’s own strength in diverse physical situations, muscular improvement), and self-confidence (satisfaction with one’s physical condition and physical fitness). The possible Likert-scale answers ranged from “totally disagree” (1) to “totally agree” (4). The reliability of the PSPP Spanish version was highly significant (α = between 0.89 and 0.69) and the internal consistency ranged from 0.70 (physical strength) to 0.80 (sport competence) [9].

The international fitness scale (IFIS) [7] was used to predict the self-reported physical fitness of adolescents in the present study. This simple self-administered scale assessed the physical fitness of the participants in a short time. Adolescents should be able to answer easy questions about their physical fitness. This instrument is composed of five questions: OPF, cardiorespiratory fitness, muscular strength, speed/agility and flexibility. However, we only focused on the OPF with the intention of knowing the perception of adolescent students in this study. The possible Likert-scale answers ranged from “very poor” (1) to “very good” (5), and the questions always invited the participants to compare the self-reported physical fitness with the physical fitness of other friends. Cohen´s kappa coefficient of test-retest in this one question showed a significant value (*p* < 0.001). The reliability of the IFIS was highly significant (α = between 0.74 and 0.82) [36] and the internal consistency ranged from 0.58 (cardiorespiratory fitness) to 0.65 (OPF) [7].

### 2.4. Procedure

All the participants were given specific information about the study (the main aim, the expected duration of the questionnaires’ interview and the procedures). In addition, participants’ parents and those responsible for the secondary school center were informed about the nature and objective of the study: body composition and psychometric variable measurement, anonymity of all responses, and non-identification of adolescent student participants. All adolescents of the present study were given two days to complete all of the measurement protocol during physical education classes. The first day, they had to complete the anthropometric measurements in order of the class list. A day after, they filled in the questionnaires related to physical self-perception (PSPP) and self-reported OPF. The instruments measuring the different variables were administered in the classroom by the researchers themselves without the teacher present. Researchers told the participants that they should be sincere with the answers of questionnaires distributed. Teachers had the possibility to obtain the results if they asked about them.

The participants of the present study were selected through the responsible secondary school and physical education teacher, who read and signed an informed consent statement before taking part in the study. The participants’ parents obtained information about the main aims of the investigation, based on the document approved by the Bioethics Committee of the University of Granada (563/CEIH/2018), and an informed consent form was signed by them. All adolescent participants in this study were treated according to the American Psychological Association (APA) guidelines with the purpose of ensuring the anonymity of the students’ responses.

### 2.5. Statistical Analyses

The normal distribution of data was analyzed using the Kolmogorov–Smirnov test. Variables studied in the present research showed a non-parametric distribution. The mean and standard deviation of the participant´s clinical characteristics (gender, BMI status, age, body weight, and body height), PSPP subdomains (physical condition, attractive body, sport competence, physical strength, and self-confidence) and self-reported OPF by IFIS were performed on student participants of the present study.

The comparison in clinical characteristics, PSPP subdomains and physical fitness categories by IFIS between boys and girls (gender) were performed by the Mann–Whitney U test; whereas the differences between clinical characteristics, PSPP subdomains, and OPF by IFIS between BMI status (underweight, normal weight and overweight/obese) were performed by the Kruskal–Wallis test. Pairwise comparisons were performed with Bonferroni’s adjustment. The magnitude of the differences in the diverse outcomes of gender and BMI status categories were calculated using the effect size [37].

The reliability assessment of the data on the five variables included in the multiple moderated mediation analysis (Figure 1) was performed by Cronbach’s alpha (α = 0.75).

The association between categorized variables (age and BMI status) and continuous variables (PSPP dimensions and OPF by IFIS) were analyzed by Spearman´s correlation coefficient. The correlation values for performance-based tests were interpreted as follows: weak or no relationship (r = 0 to 0.25), fair degree (0.25 to 0.50), and moderate-to-good (r = 0.50 to 0.75) [38].

A mediation analysis is understood as a mechanism where one mediating variable transmits the effect from an independent variable to a dependent variable, based on linear regression models. The reason not to include physical condition with the other four mediator variables is because the purpose of this PSPP subscale is similar than OPF and we have avoided an irregular consistency assessment in the mediation analysis. Moreover, it is important to highlight that OPF is a general subscale that encompasses the rest physical fitness subscales measured in the IFIS questionnaire. In order to assess whether the association between gender (independent variable) and OPF (dependent variable) was mediated by attractive body, sport competence, physical strength, self-confidence and BMI status, a multiple mediation analysis was fitted using bias-corrected bootstrapped mediation procedures [39]. Bootstrapping is a non-parametric resampling method which involves repeatedly extracting samples from the data by randomly sampling with replacement and estimating the indirect effect in each resampled data-set [40]. This multiple mediation analysis was performed using the PROCESS macro for SPSS (New York, USA), model 4 [39]. A bias corrected bootstrap based on 5000 bootstrap samples with confidence intervals (Cis, 95%) was used to test the statistical significance of the indirect and direct effects in the multiple mediation analysis. If there was not zero in the confidence intervals, the effect was considered to be significant. A statistical diagram of the indirect effect of X on Y through M and the direct effect of X on Y = c´ is shown in the hypothetical model of the Figure 1. Finally, physical condition (PSPP) was deleted from multiple mediation because OPF measures a similar subjective perception, and this fact could produce collinearity assumptions.

All statistical analyses were performed using the Statistical Package for Social Science (IBM SPSS Statistics for Windows 21.0. Armonk, NY, USA).

## 3. Results

Differences in Clinical Characteristics of the study sample, PSPP domains, and physical fitness self-reported through IFIS are specified by gender and BMI status in Table 1. Boys showed higher values than girls in body height (*p* < 0.001), all PSPP domains (*p* < 0.05) and OPF (*p* < 0.01). According to BMI status, there were significant difference between underweight, normal weight and overweight/obese in age and body weight (both, *p* < 0.001), body height (*p* < 0.01), physical condition (PSPP domain; *p* < 0.01) and OPF (IFIS category; *p* < 0.01).

Positive correlations were observed between OPF and BMI status (r = −0.324, *p* = 0.01), physical condition (r = 0.424, *p* = 0.001), attractive body (r = 0.483, *p* = 0.001), sport competence (r = 0.291, *p* = 0.01), physical strength (r = 0.455, *p* = 0.001) and self-confidence (r = 0.598, *p* = 0.001).

The multiple mediation analysis revealed that the total effect (path *c*) from gender to OPF (IFIS category) was significant, coefficient = 0.679 (95% CI: 0.271 to 1.08, *p* < 0.002) (Table 2). In addition, the specific indirect effect mediators of X (path *a*) on Y (path *b*) are showed in Table 2. With respect to the specific direct effect of each proposed mediator (path *c′*) between gender (clinical characteristic) and OPF (IFIS category), attractive body (coefficient = 0.462; 95% CI: 0.098 to 0.827, *p* < 0.013), sport competence (coefficient = 0.548; 95% CI: 0.138 to 0.959, *p* < 0.009), physical strength (coefficient = 0.421; 95% CI: 0.043 to 0.798, *p* < 0.029), self-confidence (coefficient = 0.296; 95% CI: −0.057 to 0.649, *p* < 0.099) and BMI status (coefficient = 0.602; 95% CI: 0.219 to 0.986, *p* < 0.002) were significant.

The multiple mediation estimated a completely standardized indirect effect of X on Y for attractive body (effect = 0.109), sport competence (effect = 0.066), physical strength (effect = 0.130), and self-confidence (effect = 0.193).

## 4. Discussion

The two established objectives of this study were (a) to analyze the difference of physical self-perception and self-reported physical fitness between boys and girls (gender) and BMI status and (b) to determine the mediating role of all PSPP categories (except physical condition) and BMI status in the link between gender and OPF in adolescent students (Figure 1).

Clinical characteristics showed differences between boys and girls in body height and it is important to highlight that the fact of adolescent growth of boys and girls has been discussed as 1976 [41]. The average body height in this study is in line with a multilevel longitudinal analysis of sex differences in height gain and growth were Japanese boys showed higher body height than girls. However, the girls gain peaked approximately two years earlier than boys [42]. Body height is a specific indicator of puberty. It is related to the effects of this period and is generally associated to body height growth differences between boys and girls [43,44], with a higher growth velocity for boys [45]. Moreover, prospective studies about prepubertal body composition have concluded that female pubertal development is intermittent [3,4,5,6,7,8,9,10,11,12,13,14,18,19,20,21,22,36]. Equally, age as an early pubertal marker could explain the relationship between body composition characteristics and pubertal development results (increasing of height, muscle, and bone mass, etc.). Girls experience body weight changes only after menarche with a higher BMI. These changes cannot be considered to be determinant of earlier puberty onset. Higher prepubertal BMI may be associated with earlier menarche in girls [46], while there is not sufficient data about an increase of BMI after the beginning of the puberty in boys [47]. Li et al.’s study [46] concludes that further research is necessary to clarify whether a critical time window exists to explain an increase of body weight levels in early puberty.

In this study, important results showed that positive attractive body, sport competence, physical strength, and self-confidence (all, PSPP subscales) were significant mediators in the link between gender and OPF. Adolescent students of the present study experienced differences between genders in those PSPP subscales. A multitude of reasons maybe attributed to the observed differences in the physical self-concept of boys and girls. In this sense, it must be highlighted that girls normally show a less favorable relationship with the five PSPP subscales than boys, specifically in physical condition, sport competence, and attractive body [1,9,15,48]. According to the last PSPP subscale, attractive body plays an important role in youth because the obsession with perfection of the body is constant in their daily life. In fact, the fascination for beauty is common in all ages and sectors of society and not only characteristic of young people [18]. The feeling of beauty and satisfaction with one’s own body may accompany the growth and maturation of both boys and girls since early childhood. Youngsters undergo physical and cognitive changes just before the beginning of the adolescence that influence their personal and social identity construction process [1].

Our findings coincide with Murcia and Cervelló’s [9] statement that boys normally feel stronger physical self-confidence in their self and their attractiveness perception is higher than in girls. This fact could be the result of regular PA’s effects on male attractive body and the explanation why boys behave with higher attractive body self-confidence than girls. This is a only an hypothesis, since the PA level has not been measured in the present study; although a number of studies confirm this relationship in adolescents [1,2,9,49,50]. Moreover, attractive body showed a positive mediation in the link between boys and OPF. A possible reason for this is that they have a more favorable self-perception regarding their physical self-concept than girls [1,35]. In particular, girls very often report low general physical self-perceptions associated with a negative body image with social physical anxiety or depression [51]. Consequently, the female perception of OPF might be mediated by a low attractive body concept of their self. However, attractive body could not be affected directly by the practice of PA [52] because changes of PA in adolescent girls are mainly predicted by the physical condition perceived [49] instead of cognitive variables such as body image self-perception. This means that we must be cautious with the findings about the mediation of attractive body between gender and OPF. Differences by biological characteristics between boys and girls must be also taken into account as a possible explanation of the attractive body mediation on OPF, specifically in girls. Puberty signs appear before in girls than boys [46], with numerous physiological modification as breast and pubic hair development, facial features, etc. [46,53]. Moreover, some body symmetry and hormone signals are often perceived as attractive or unattractive among young children and adolescents [54]. Thus, girls could try to hide their physical changes in physical tasks as physical exercise or sport and consequently, showing lower OPF because they feel less attractive.

Similarly, sport competence perceived by participants of this study follow results similar to the attractive body mediation analysis with respect to the relationship between gender and OPF. A previous study with Spanish students showed greater sport competence perception in boys than girls and an association between sport competence and physical strength with a general fitness [50]. In another study with Spanish adolescents, it was found that male participants obtained higher scores in attractive body than female participants [9]. Those studies showed similar results than our study. However, the mediation role of sport competence has an impact on OPF for boys. The impact of sport on physical and perceived social competence might explain the improvement of physical health and athletic competence [19]. Therefore, boy participants in this study could perceive a higher OPF score because their sport abilities, motivation, or satisfaction built a stronger physical self-concept than girls and therefore a greater self-perception of physical fitness in general. In contrast, girls could differ from boys in sport competence perception due to difference between genders according to the perceptions of social competence in PA practice and sport [19]. Regardless, it is not easy to discuss the different effects of gender on OPF perception through sport competence because the differences of Sport self-perception between boys and girls may decrease in later adolescence [55].

Regarding the perception of strength in adolescents, the physical self-concept is always associated with Physical Strength perception in both boys and girls whether an adequate physical fitness level exists [21]. However, we must be cautious because the adolescents begin to recognize different physical self-concepts when they are growing up and this fact is accompanied of self-esteem declination or underestimation [56]. On the other side, adolescents with regular frequency of PA practice report higher self-confidence, autonomy, self-motivation and physical self-concept overestimation and this outcome have to be also interpreted with caution [57]. Muscularity and physical strength is typically linked with boys instead of girls [24]. Related to the latter, boys normally perceive higher physical strength than girls and show significant association between physical strength and general fitness [50]. On the contrary, adolescent girls reveal a weak association between physical strength perception and physical self-worth [25]. Likewise, the effect of physical strength on OPF is preceded by studies where the correlation between physical strength and general perception of physical fitness is significant [58], especially in male children and adolescents [50]. Given that our findings about the physical strength mediation role follows the previous studies cited about the effects gender and physical strength and physical strength with OPF, we could be in position to affirm that boys experience greater physical strength perception because of they perceive stronger physical self-confidence, and this fact produces higher self-reported OPF.

The intention to be physically active is important in adolescents, demonstrating the relevance of self-confidence and perceived physical fitness whether there is a wish to practice PA or a sport [59]. Self-confidence is the most important physical exercise and fitness predictor when there is an empowerment of ego due to the increase of the physical fitness concept [12]. In general, self-confidence has been shown to be an essential factor of mental health [60]. In particular, girls suffer lower self-confidence than boys, and these mental symptoms are related to psychological disorders [60]. That is why girls should be more active and practice PA with the aim of increasing their psychosocial well-being [61], preserve mental health, and strengthen self-confidence. Otherwise, when the measurement of self-confidence perception is used with male adolescents, it is normal to find outcomes with greater levels of physical self-confidence [62] and social interactions [57]. Boys normally rate themselves with higher self-confidence than girls and higher participation in PA during their daily life [63]. Thus, boys could be more active than girls, and therefore the greater perceived self-confidence would have an impact in OPF with a higher physical fitness perception. On the other hand, greater self-confidence is normally linked to different components of health-related physical fitness both in boys and girls, being an important pillar for a physically active life [64]. In general, the OPF shows a positive relationship with cognitive factors in physically active students regardless of gender within the school context. In addition, cognitive factors may predict a better OPF in physical education settings or in any other context [65]. However, according to the authors of the construct validity and test-retest reliability of the IFIS questionnaire in Spanish children [3], it is not strange to find differences between boys and girls in the main physical fitness components (cardiorespiratory fitness, muscular strength, speed/agility and flexibility). In accordance with the discussed results in the relationship between gender and OPF and mediation by five variables (attractive body, sport competence, physical strength, self-confidence and BMI status), we can summarize the main factors on OPF between boys and girls as the physical self-concept perceived, the satisfaction with the one’s own body, the perceived social competence, and an adequate physical fitness level perceived.

## 5. Conclusions

The present study highlights the mediation role of four physical self-perception subscales (attractive body, sport competence, physical strength, and self-confidence) in the direct effect of gender (boys and girls) on OPF perception of adolescent students. Boys perceived greater physical self-confidence in those four subscales and also OPF than girls. Thus, we can confirm high inequality between the genders of our secondary school participants. Considering the importance of physical self-perceptions studied in the mediation of gender on OPF, strategies to improve the self-perception of adolescents should be considered, specifically in female adolescents. Moreover, the association between the regular practice of PA and greater physical self-perception is obvious. As a consequence, higher levels of PA in adolescents are clearly related to higher greater OPF perception. Finally, it should be noted that girls are a risk group because they report low physical self-confidence with their respective insecurity feelings and psychological disorders. Strategies focused to improve self-confidence and physical self-perception are necessary in children and adolescent students.

Future research on adolescent students with similar characteristics to those evaluated in this study should focus on the improvement of physical self-perception by greater PA practice in adolescents, specifically in female adolescents. Poor physical self-perception in attractive body, sport competence, physical strength, and self-confidence could have a negative influence on the mental and physical development of girls, and consequently the OPF perceived is not going to be good.

There are some limitations in this study that could influence in the interpretations of the main outcomes. The sample of secondary school students in our research is not large and the results could be stronger with a larger sample. Moreover, objective OPF as dependent variable would be necessary in further studies where the physical self-perception act as mediator with respect to gender or another independent variable.

Despite the limitations, the present study contributes to the understanding of the relationships between gender and OPF through attractive body, sport competence, physical strength, and self-confidence (physical self-perception) as mediators.

## Figures and Tables

**Table 1:** Clinical Characteristics, physical self-perception profile (PSPP) domains and overall physical fitness through international fitness scale (IFIS) of adolescent students by gender and body mass index (BMI) status.

Variables	Gender M (SD)				BMI Status M(SD)					
	Girls (N = 44)	Boys (N = 41)	*p*-value ^†^	Effect Size	Underweight (N = 19)	Normal Weight (N = 53)	Overweight/Obese (N = 13)	*p*-value ^‡^	Effect Size	Total (N = 85)
Age (years)	14.43 (1.60)	14.66 (1.69)	0.422	−0.13	13.47 (1.83) ^a^	15.11 (1.38) ^a,b^	13.77 (1.30) ^b^	0.001	0.35	14.54 (1.64)
Body Weight (kg)	58.20 (12.43)	61.68 (12.57)	0.128	−0.27	43.37 (4.51) ^a,b^	61.58 (7.85) ^a,c^	77.08 (6.33) ^b,c^	0.001	0.39	59.88 (12.55)
Body Height (meters)	1.62 (0.07)	1.69 (0.09)	0.001	−0.86	1.58 (0.08) ^a,b^	1.67 (0.08) ^a^	1.67 (0.05) ^b^	0.004	0.39	1.65 (0.09)
PSPP (range 1–4)										
Physical Condition	3.00 (1.26)	3.53 (.92)	0.01	−0.59	3.57 (0.84) ^a^	3.32 (1.11) ^b^	3.15 (1.28) ^a,b^	0.005	0.39	4.07 (1.15)
Attractive Body	3.00 (1.31)	3.43 1.07)	0.05	−0.45	3.53 (0.91)	3.21 (1.18)	3.38 (1.61)	0.138	0.38	4.01 (1.23)
Sport Competence	2.56 (1.45)	3.12 (1.17)	0.05	−0.53	3.20 (1.15)	2.76 (1.38)	3.23 (1.53)	0.231	0.38	3.54 (1.36)
Physical Strength	3.04 (1.34)	3.57 (.92)	0.01	−0.57	3.49 (1.01)	3.34 (1.12)	3.54 (1.62)	0.320	0.38	4.12 (1.19)
Self-Confidence	2.56 (1.56)	3.23 (1.18)	0.01	−0.66	3.24 (1.39)	2.86 (1.39)	3.31 (1.79)	0.346	0.38	3.65 (1.46)
IFIS (range 1–5)										
Overall Physical Fitness	3.23 (1.01)	3.90 (0.86)	0.01	−0.71	4.00 (0.88) ^a^	3.58 (0.90) ^b^	2.77 (1.10) ^a,b^	0.004	0.39	3.55 (0.99)

Note: Values are means (standard deviation); † *p* values calculated by the Mann–Whitney U test between boys and girls; ^‡^ *p* values calculated by the Kruskal–Wallis test between BMI status. Effects size statistics between boys/girls and BMI status are expressed with Cohen’s d (effect size-*r).* ^a,b,c^ Common superscripts in the same row indicate a significant difference (*p* < 0.05) between the groups with the same letter. Pairwise comparisons in BMI were performed with Bonferroni’s adjustment.

**Table 2:** Indirect effect of gender (male/female) on overall physical fitness of adolescent students through attractive body, sport competence, physical strength, self-confidence, and body mass index (BMI) status.

Mediator	Effect of X on M (a^1^–a^5^)	*p*-value	SE	Effect of M on Y(b^1^–b^5^)	*p*-value	SE	Bootstrap Estimate	*SE*	BCa 95% CI	
									Lower	Upper
Attractive Body	0.551	0.039	0.263	0.394	0.001	0.074	0.217	0.121	0.021	0.501 ^†^
Sport Competence	0.699	0.018	0.291	0.187	0.015	0.075	0.131	0.086	0.012	0.353 ^†^
Physical Strength	0.672	0.001	0.253	0.384	0.001	0.079	0.258	0.111	0.082	0.519 ^†^
Self-Confidence	0.953	0.002	0.299	0.402	0.001	0.601	0.383	0.131	0.163	0.683 ^†^
BMI status	−0.758	0.322	0.761	−0.101	0.001	0.027	0.077	0.081	−0.059	0.268

*Note*. ^†^ Significant indirect effect of X on Y through M. SE = standard error; BCa = bias corrected and accelerated.
